# How Long to Starve

**Published:** 1871-04

**Authors:** 


### HOW LONG TO STARVE.

A MAN will die for want of air in five minutes, for want of sleep in ten days, for want of water in a week, for want of food at varying intervals, dependent on constitution, habits of life, and the circumstances of the occasion. Instances have been given where persons have been said to live many weeks without eating a particle of food; but when opportunities have been offered for a fair investigation of the case, it has been invariably found that a weak and wicked fraud has been at the bottom of it.

On the 28th of August, the captain of a Boston whaler was wrecked. For eight days he could not get a drop of water, nor a particle of food. On the day of the wreck he weighed a hundred and ninety pounds; when rescued, he weighed one hundred pounds. A tea-spoonful of brandy was given to each sailor; but before they could be taken aboard the vessel which saved them, they became unconscious, and remained so for two days, but all eventually recovered. Many persons have been killed by eating too much after having fasted for a long time; the safe plan of procedure, and which every reader should bear in mind, is to feel the way along, as persons who are travelling in the dark and fear a precipice ahead; there can be no one rule given, because there are so many modifying circumstances. Give a tea-spoonful of hot drink at a time, and if no ill result, repeat in five minutes, and the same amount of soft food, boiled rice, or softened bread, or soup, or gruel; for the stomach is itself as weak as the sufferer in proportion, and can only manage a very small amount of food.

Wading in water, or keeping the clothing saturated with water, even if it is sea-water, sensibly abates the horrors of thirst.

